# Genome-Wide Association Scan Identifies a Risk Locus for Preeclampsia on 2q14, Near the Inhibin, Beta B Gene

**DOI:** 10.1371/journal.pone.0033666

**Published:** 2012-03-14

**Authors:** Matthew P. Johnson, Shaun P. Brennecke, Christine E. East, Harald H. H. Göring, Jack W. Kent, Thomas D. Dyer, Joanne M. Said, Linda T. Roten, Ann-Charlotte Iversen, Lawrence J. Abraham, Seppo Heinonen, Eero Kajantie, Juha Kere, Katja Kivinen, Anneli Pouta, Hannele Laivuori, for the FINNPEC Study Group, Rigmor Austgulen, John Blangero, Eric K. Moses

**Affiliations:** 1 Department of Genetics, Texas Biomedical Research Institute, San Antonio, Texas, United States of America; 2 Department of Perinatal Medicine/Department of Obstetrics and Gynaecology, Royal Women's Hospital and University of Melbourne, Parkville, Victoria, Australia; 3 Department of Cancer Research and Molecular Medicine, Norwegian University of Science and Technology, Trondheim, Norway; 4 The School of Biomedical Biomolecular and Chemical Sciences, The University of Western Australia, Perth, Western Australia, Australia; 5 Department of Obstetrics and Gynecology, Kuopio University Hospital, and University of Eastern Finland, Kuopio, Finland; 6 Department of Chronic Disease Prevention, Diabetes Prevention Unit, National Institute for Health and Welfare, Helsinki, Finland; 7 Children's Hospital, Helsinki University Central Hospital and University of Helsinki, Helsinki, Finland; 8 Department of Biosciences and Nutrition, and Science for Life Laboratory, Karolinska Institutet, Stockholm, Sweden; 9 Folkhälsan Institute of Genetics, Helsinki, Finland; 10 Haartman Institute, Department of Medical Genetics, University of Helsinki, Helsinki, Finland; 11 Wellcome Trust Sanger Institute, Wellcome Trust Genome Campus, Hinxton, Cambridge, United Kingdom; 12 Department of Children, Young People and Families, National Institute for Health and Welfare, Oulu, Finland; 13 Research Programs Unit, Women's Health, University of Helsinki, Helsinki, Finland; 14 The Centre for Genetic Epidemiology and Biostatistics, The University of Western Australia, Perth, Western Australia, Australia; The Children's Hospital of Philadelphia, United States of America

## Abstract

Elucidating the genetic architecture of preeclampsia is a major goal in obstetric medicine. We have performed a genome-wide association study (GWAS) for preeclampsia in unrelated Australian individuals of Caucasian ancestry using the Illumina OmniExpress-12 BeadChip to successfully genotype 648,175 SNPs in 538 preeclampsia cases and 540 normal pregnancy controls. Two SNP associations (rs7579169, p = 3.58×10^−7^, OR = 1.57; rs12711941, p = 4.26×10^−7^, OR = 1.56) satisfied our genome-wide significance threshold (modified Bonferroni p<5.11×10^−7^). These SNPs reside in an intergenic region less than 15 kb downstream from the 3′ terminus of the Inhibin, beta B (*INHBB*) gene on 2q14.2. They are in linkage disequilibrium (LD) with each other (r^2^ = 0.92), but not (r^2^<0.80) with any other genotyped SNP ±250 kb. DNA re-sequencing in and around the *INHBB* structural gene identified an additional 25 variants. Of the 21 variants that we successfully genotyped back in the case-control cohort the most significant association observed was for a third intergenic SNP (rs7576192, p = 1.48×10^−7^, OR = 1.59) in strong LD with the two significant GWAS SNPs (r^2^>0.92). We attempted to provide evidence of a putative regulatory role for these SNPs using bioinformatic analyses and found that they all reside within regions of low sequence conservation and/or low complexity, suggesting functional importance is low. We also explored the mRNA expression in decidua of genes ±500 kb of *INHBB* and found a nominally significant correlation between a transcript encoded by the *EPB41L5* gene, ∼250 kb centromeric to *INHBB*, and preeclampsia (p = 0.03). We were unable to replicate the associations shown by the significant GWAS SNPs in case-control cohorts from Norway and Finland, leading us to conclude that it is more likely that these SNPs are in LD with as yet unidentified causal variant(s).

## Introduction

Preeclampsia is a common and serious complication of human pregnancy affecting 3–5% of all primigravid women [1]–[3]. Delivery of the fetus and placenta is the only intervention for adequate resolution of severe symptoms. It is a major cause of maternal mortality in developing countries, accounting for 50,000 maternal deaths yearly [4]. The maternal and fetal morbidity and mortality associated with preeclampsia and in particular with the adverse consequences of pre-term delivery are a major health burden in the developed world [5]–[7].

The pathophysiology of preeclampsia is thought to involve two main stages [8], [9]. In stage one abnormal fetal-derived cytotrophoblast invasion in the uterine wall in early pregnancy is associated with failed remodeling of the maternal spiral arteries perfusing the placenta. This is thought to be a ‘root’ cause. As a result of hypoxia and/or oxidative stress to the placenta there is release of syncytiotrophoblast-derived factors into the maternal circulation that give rise to the second stage of the maternal syndrome. The known placental factor of most relevance to this second stage is the soluble receptor for vascular endothelial growth factor, sVEGFR-1, also called sFlt-1. When present in excess, as in preeclampsia, sFlt-1 binds to, and activates, VEGF, a key survival factor for endothelium [10], and thereby induces systemic endothelial dysfunction.

The principal diagnostic features of preeclampsia are new onset hypertension and proteinuria after 20 weeks gestation [11]. The hypertension is now recognized to be secondary to diffuse endothelial dysfunction [12], and the proteinuria is associated with glomerular endotheliosis [10], [13]. Preeclampsia is therefore primarily characterized by endothelial dysfunction, which is also one of the principal pathogenic mechanisms in atherosclerotic vascular diseases such as coronary artery disease and stroke. Consistent with their shared pathogenesis, atherosclerosis and preeclampsia share many common risk factors including hypertension, obesity, insulin resistance, diabetes mellitus, metabolic syndrome, general inflammation, thrombophilia, and family history [14]. A history of preeclampsia increases the risk of future hypertension, ischemic heart disease, stroke and venous thromboembolism. This is true especially for women with a history of early-onset preeclampsia (<34 weeks gestation) than those women who have preeclampsia at term [15]. A popular theory is that pregnancy provides a metabolic stress test to unmask underlying risk of cardiovascular disease [16].

These data have led several investigators to speculate [17], [18] that the genetic risk factors for preeclampsia will also be relevant to cardiovascular disease, providing increased impetus and justification for their discovery [19], [20]. By far the most effort to date has been focused on candidate genes, primarily those for which a plausible role in the known underlying pathophysiology could be argued, and in particular blood pressure regulation, endothelial dysfunction, lipid metabolism, thrombophilia, placental development and function, and the inflammatory response [21]. There have been many nominal associations reported with a lack of reproducibility a common theme, in many cases most likely due to a lack of uniformity in diagnosis and underpowered study designs. In our attempts to identify risk factors for preeclampsia we have primarily focused on positional cloning strategies, making no *a priori* assumptions about the nature of genes involved. We initially performed genome-wide linkage mapping studies in multiple affected families from Australia and New Zealand, identifying putative susceptibility loci on chromosomes 2q22, 5q and 13q [22], [23], with several plausible positional candidate susceptibility genes identified, including the activin receptor gene *ACVR2A* on 2q22 [24], [25], the aminopeptidase gene *ERAP2* on 5q [26], and the cytokine encoding *TNFSF13B* gene on 13q [27]. We now report on our continued positional cloning efforts using genome-wide association mapping in a large Caucasian case-control cohort from Australia. We herein report a significant novel SNP association on chromosome 2q14.2, close to the Inhibin, beta B (*INHBB*) gene.

## Results

### Australian GWAS

#### Genome-wide association with preeclampsia

**Figure 1:**
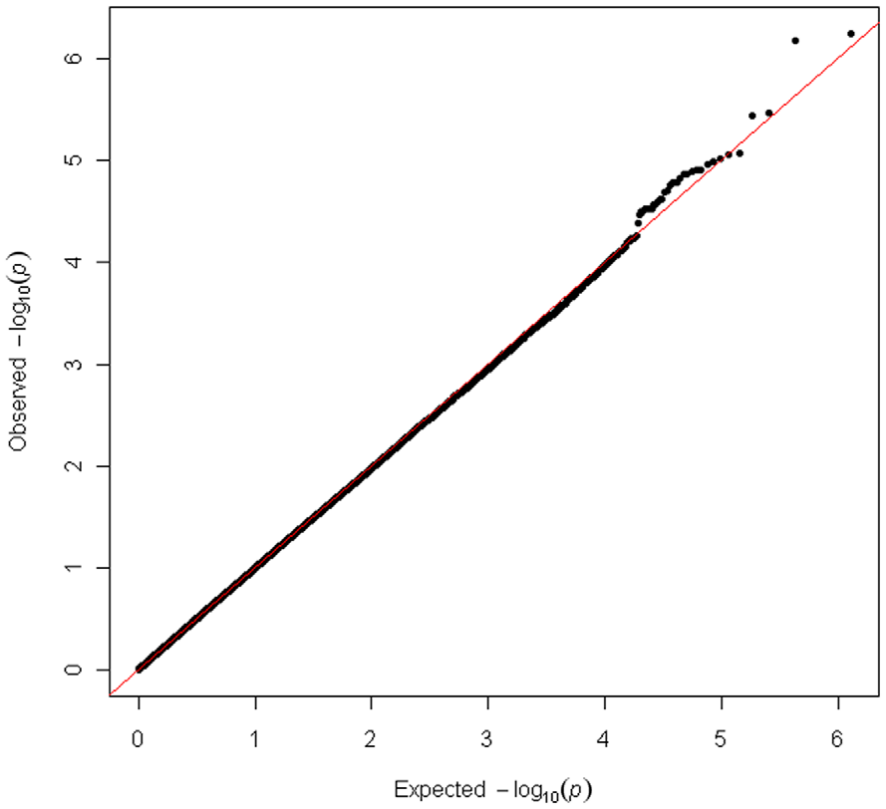
Quantile-quantile (Q-Q) plot of the observed GWAS p-values (−log_10_P).

**Figure 2:**
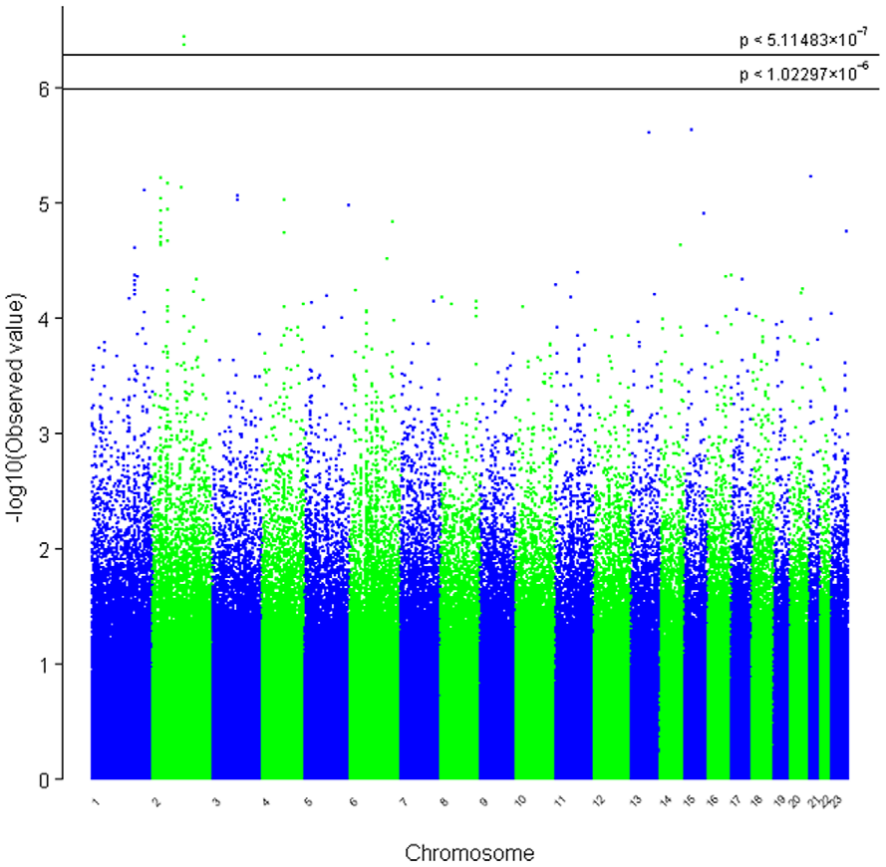
The genome-wide distribution of asymptotic p-values for each of the quality control filtered SNPs in the Australian cohort (n = 648,175). Our adjusted genome-wide significant and suggestive thresholds were set at p<5.11483×10^−7^ and p<1.02297×10^−6^, respectively.

**Figure 3:**
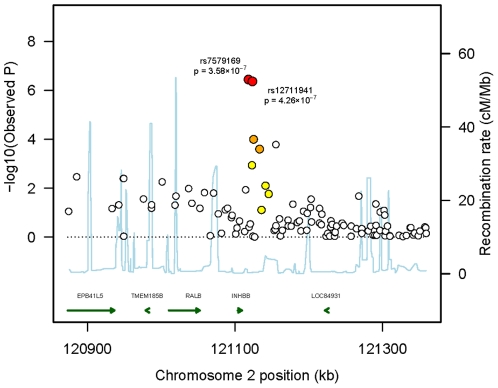
Association plot of the chromosome 2 region reaching genome-wide significance with preeclampsia susceptibility (rs7579169±250 kb). Observed p-values are plotted as −log10 values as a function of the SNPs physical location (NCBI Build 37.2). Estimated recombination rates were extracted from HapMap data. The local linkage disequilibrium structure is based on the observed allele frequency data in the Australian cohort (red dots, r^2^≥0.8; orange dots, 0.5≤r^2^<0.8; yellow dots, 0.2≤r^2^<0.5; clear dots, r^2^<0.2). Gene annotations were obtained from the UCSC genome browser (Human, Feb. 2009 [GRCh37/hg19]).

In the set of 1,078 unrelated Australian samples (538 preeclampsia cases, 540 normal pregnancy controls) that passed our quality control criteria, the observed distribution of p-values for 648,175 successfully genotyped SNPs exhibited minimal deviation from the expected distribution (Figure 1). As such, this indicates minimal test statistic bias or underlying population structure (λ = 1.002). The −log10 transformation of observed p-values across the genome are displayed in Figure 2 and SNPs with a p-value of 10^−6^ or less are presented in Table 1. By accounting for the extent of SNP linkage disequilibrium (LD), per chromosome, the number of independent SNPs (SNP_INDEP_) across the genome were estimated (Table S1). The estimated number of independent SNPs, specific to the Australian case-control cohort, was used in a modified Bonferroni procedure to generate an adjusted target alpha level (0.05/SNP_INDEP_). The two most significant SNP associations satisfied our genome-wide significance threshold (modified Bonferroni p<5.11×10^−7^) (Figure 2). The SNP showing the strongest association (rs7579169; p = 3.58×10^−7^, OR = 1.57, MAF_(cases)_ = 0.447, MAF_(controls)_ = 0.340) is intergenic and resides ∼8.3 kb downstream from the 3′ terminus of the *INHBB* gene on chromosome 2q14.2. The next strongest association is also for an intergenic SNP (rs12711941; p = 4.26×10^−7^, OR = 1.56, MAF_(cases)_ = 0.448, MAF_(controls)_ = 0.342) downstream (∼13.5 kb) from the 3′ terminus of the *INHBB* gene. Both of these SNPs are strongly correlated with each other (r^2^ = 0.92), but not (r^2^<0.80) with any other genotyped SNP ±250 kb (Figure 3). The SNP chip used in this study accommodated two SNPs within the *INHBB* gene locus itself, a nearGene-5 (rs13419301) and an intronic (rs11902591) SNP. Both of these *INHBB* locus SNPs did not reach nominal significance (p = 0.43 and p = 0.22, respectively), nor were they correlated with rs7579169 (r^2^ = 0.03 and r^2^ = 0.02, respectively). The sample genotype success rates for rs7579169 and rs12711941 were 0.9981 and 1.0, respectively. Furthermore, sample genotype concordance rates for rs7579169 and rs12711941 genotyped on both the Illumina and Sequenom platforms (see methods) were 0.987 and 0.993, respectively.

**Table 1:** SNP associations with preeclampsia (p≤10^−6^).

Chr	SNP	bp^1^	Function	Gene	Alleles^2^	MAF_(cases)_	MAF_(controls)_	P-value	OR^3^ (95% CI)
2	rs7579169	121118124	intergenic		C/T	0.4471	0.3401	3.58×10^−7^	1.57 (1.32–1.87)
2	rs12711941	121123383	intergenic		G/T	0.4482	0.3420	4.26×10^−7^	1.56 (1.31–1.86)
15	rs2453274	44774202	intronic	*CTDSPL2*	C/T	0.0462	0.0989	2.32×10^−6^	0.44 (0.31–0.62)
13	rs12431203	77488736	intergenic		G/A	0.2061	0.1303	2.45×10^−6^	1.73 (1.38–2.18)
21	rs2826538	22188735	intergenic		T/C	0.1954	0.2782	5.93×10^−6^	0.63 (0.52–0.77)
2	rs9332419	26322040	intronic	*RAB10*	G/A	0.3562	0.4518	6.17×10^−6^	0.67 (0.56–0.80)
2	rs4952830	46746963	UTR-5	*ATP6V1E2*	A/G	0.3343	0.4284	6.74×10^−6^	0.67 (0.56–0.80)
2	rs6542736	108415702	intergenic		G/A	0.4094	0.3167	7.31×10^−6^	1.50 (1.25–1.79)
1	rs6660579	224916592	intronic	*CNIH3*	C/T	0.1558	0.0924	7.80×10^−6^	1.81 (1.39–2.36)
3	rs2279720	87276699	synonymous	*CHMP2B*	G/A	0.0592	0.1128	8.66×10^−6^	0.49 (0.36–0.68)
3	rs1044499	87299075	synonymous	*CHMP2B*	A/C	0.0592	0.1128	8.66×10^−6^	0.49 (0.36–0.68)
2	rs11126375	26344787	intronic	*RAB10*	A/G	0.1645	0.2412	9.13×10^−6^	0.62 (0.50–0.77)
4	rs7677523	98965696	intronic	*C4orf37*	T/C	0.0833	0.0379	9.51×10^−6^	2.31 (1.58–3.37)
3	rs17024019	87257423	intergenic		A/G	0.0601	0.1137	9.57×10^−6^	0.50 (0.36–0.68)

1 Physical coordinate based on NCBI reference assembly build 37.2.

2 Major allele/minor allele.

3 Odds ratio for the minor allele.

#### HapMap CEU proxy SNPs

To investigate other potential proxy SNPs to rs7579169, we used the latest (19-Apr-2009) HapMap CEU linkage disequilibrium (LD) data arising from phases I+II+III (rel #27, NCBI B36). Based on current HapMap parameters the search for SNPs flanking rs7579169 was restricted to ±200 kb. One additional proxy SNP (rs7576192) was identified to be strongly correlated with rs7579169 in the CEU genotype data (r^2^ = 1) (Table S2). The rs7576192 SNP also resides downstream from the 3′ terminus of the *INHBB* gene and is 93 bp from rs7579169 (Table S2). In the CEU samples, rs7576189 is also strongly correlated with rs12711941 (r^2^ = 0.96) and not correlated with rs13419301 (r^2^ = 0.04) or rs11902591 (r^2^ = 0.03) (Table S2). These data are concordant with our Australian GWAS cohort. In addition, a second *INHBB* nearGene-5 SNP (rs7578624) genotyped in the CEU samples was not correlated with rs7579169 (r^2^ = 0.02) (Table S2).

#### SNP degree of dominance

We investigated the mode of inheritance of our top two SNP associations by estimating the degree of dominance index (*h*) [28]. We report negligible deviation from additivity and hence, a significant additive effect for both the rs7579169 (*h* = −0.04) and rs12711941 (*h* = −0.13) SNPs. The application of PLINK's [29] –model option confirms a stronger additive effect than a dominant or recessive effect for either SNP (data not shown).

### *INHBB* locus sequencing

In an effort to identify other potentially causal variants at the *INHBB* locus we re-sequenced the entire *INHBB* structural gene (NM_002193.2), ∼2.5 kb upstream of the *INHBB* translation start site and ∼3 kb downstream of the *INHBB* STOP codon. We also sequenced a region flanking rs7579169 (∼2.2 kb upstream and ∼0.6 kb downstream) that exhibited evolutionary conservation amongst the rhesus monkey (*Macaca mulatta*), dog (*Canis familiaris*) and mouse (*Mus musculus*). Sequencing experiments were conducted in 96 individuals from the Australian GWAS cohort (48 preeclampsia cases, 48 normal pregnancy controls). These individuals were selected on the basis of carrying two copies of the rare allele at both the rs7579169 and rs12711941 SNP loci. A total of 19 SNPs (9 known, 10 novel) plus six novel deletions were identified in our Australian cohort subset (Table 2). Due to the rare ‘T’ allele for rs7579169 being concordant with our reference sequence template, this SNP locus was not highlighted in our list of identified *INHBB* locus variants.

**Table 2:** *INHBB* locus variants identified in a sample of the Australian cohort (n = 96).

Variant	bp	Function	Alleles^1^	MAF_(cases)_	MAF_(controls)_	HWEp^2^	P-value
ss469271203	121101331	nearGene-5	G/A	0.0277	0.0133	1.26×10^−9^	0.0201
ss469271213	121101394	nearGene-5	AAG/-	0.0009	0.0019	1.0	1.0*
ss469271214	121101650	nearGene-5	AGCTGG/-	Failed assay			
ss469271215	121101736	nearGene-5	CGCCGCAGCGCC/-	Failed assay			
rs7578624	121102479	nearGene-5	C/G	0.0487	0.0427	1.0	0.5080
rs13419301	121102572	nearGene-5	T/C	0.0580	0.0655	0.28	0.4803
ss469271216	121104688	intronic	TG/-	Failed assay design			
ss469271204	121105292	intronic	C/G	0.0066	0.0038	1.0	0.5478*
rs11902591	121106003	intronic	A/G	0.0673	0.0731	0.81	0.6068
rs4328642	121106850	synonymous	C/T	0.0328	0.0363	1.0	0.6662
ss469271205	121106946	synonymous	G/A	0.0009	0.0	1.0	0.4929*
ss469271217	121107784	UTR-3	AGTC/-	0.0009	0.0	1.0	0.4962*
ss469271206	121107831	UTR-3	G/A	0.0009	0.0	1.0	0.4929*
rs45624437	121108182	UTR-3	T/C	0.0162	0.0083	1.0	0.0987
ss469271207	121108506	UTR-3	G/A	0.0	0.0009	1.0	1.0*
ss469271208	121108585	UTR-3	T/C	0.0087	0.0009	0.02	0.0111*
rs57802235	121109444	nearGene-3	G/A	0.0416	0.0466	0.72	0.5788
rs7568413	121109612	nearGene-3	C/T	Non-polymorphic^3^			
ss469271209	121109737	nearGene-3	C/T	0.0048	0.0019	1.00	0.2813*
rs10183524	121109878	nearGene-3	G/A	0.0491	0.0493	0.74	0.9829
ss469271210	121110151	nearGene-3	G/A	0.0039	0.0	1.0	0.0602*
ss469271218	121116483	intergenic	A/-	0.0028	0.0009	1.0	0.6247*
ss469271211	121116625	intergenic	G/A	0.0009	0.0	1.0	1.0*
ss469271212	121116672	intergenic	C/T	0.0029	0.0009	1.0	0.3709*
rs7576192	121118031	intergenic	G/A	0.4499	0.3392	0.13	1.48×10^−7^

Identified variants were genotyped in Australian individuals passing GWAS quality control criteria (n = 1,078). Novel variants submitted to dbSNP are assigned with their ‘ss’ submission ID number.

* Fisher's exact test p-value.

1 Major allele/Minor allele.

2 Hardy-Weinberg equilibrium p-value.

3 Preeclampsia dataset allele discordant to reference template allele.

### *INHBB* variant genotyping and association analysis

Of the 25 *INHBB* locus variants identified by re-sequencing, 21 were successfully genotyped with a mean (range) genotyping success rate of 0.975 (0.960–0.999) (Table 2). Of the remaining variants, one deletion failed assay design, two deletions failed the assay and one SNP was non-polymorphic due to a discordance between the preeclampsia data set allele and the reference template allele. We observed nominal genetic associations for a novel SNP 2,434 bp upstream of the *INHBB* translation start site (ss469271203; p = 0.02, MAF_(cases)_ = 0.028, MAF_(controls)_ = 0.013) and for a rare novel SNP within *INHBB*'s 3′UTR (ss469271208; p = 0.01, MAF_(cases)_ = 0.009, MAF_(controls)_ = 0.001) (Table 2). A genome-wide significant association was observed for another intergenic SNP residing downstream from the 3′ terminus of the *INHBB* gene (rs7576192; p = 1.48×10^−7^, OR = 1.59, MAF_(cases)_ = 0.449, MAF_(controls)_ = 0.339) (Table 2). SNP rs7576192 is in close proximity to, and strongly correlated with the two significant GWAS SNPs (r^2^>0.92) (Table S3). The genotypic correlation data between rs7576192, rs7579169 and rs12711941 in our Australian cohort is concordant with the reported HapMap CEU data.

### Analysis of gene expression at the *INHBB* locus

To investigate whether expression of the *INHBB* locus was correlated with preeclampsia or the significant GWAS SNPs exhibited regulatory potential, total RNA from decidual basalis tissue of 60 individuals from the Australian case-control cohort (25 preeclampsia cases and 35 normal pregnancy controls) were hybridized onto Illumina's HumanHT-12 v4 Expression BeadChips. Our analysis of differential mRNA expression at the *INHBB* locus (ILMN_1685714) was extended ±500 kb of *INHBB*, also bringing into consideration several other genes, including *PTPN4* (ILMN_1793549), *EPB41L5* (ILMN_1770245, ILMN_2043306), *TMEM185B* (ILMN_2231020, ILMN_2231021), *RALB* (ILMN_1676358) and *GLI2* (ILMN_1727577). One transcript (ILMN_2043306) was not significantly detected (FDR p>0.05) and an additional transcript (ILMN_1727577) had a mean expression level consistent with background noise (mean average raw signal <50) (Table 3). We observed a nominally significant correlation with the ILMN_1770245 (*EPB41L5*) transcript and preeclampsia (p = 0.03) (Table 3). The independent addition of our GWAS SNPs (rs7579169, rs12711941) yielded neither as significant predictor variables in the ‘transcript∼preeclampsia’ regression model (p>0.05) (Table 3).

**Table 3:** Gene expression results of the *INHBB* structural locus ±500 kb.

Gene	Transcript	n^1^	p^2^	FDR p^3^	MARS^4^	PE^5^	rs7579169^6^	rs12711941^7^
*PTPN4*	ILMN_1793549	59	1.7×10^−77^	2.5×10^−76^	102.8	0.99	0.37	0.53
*EPB41L5*	ILMN_1770245	58	1.9×10^−74^	1.1×10^−73^	89.6	0.03	0.90	0.57
	ILMN_2043306	3	0.57	0.91	1.0	NA^8^	NA^8^	NA^8^
*TMEM185B*	ILMN_2231020	59	1.7×10^−77^	2.5×10^−76^	278.6	0.48	0.25	0.19
	ILMN_2231021	58	1.9×10^−74^	1.1×10^−73^	141.3	0.29	0.22	0.06
*RALB*	ILMN_1676358	58	1.9×10^−74^	1.1×10^−73^	1,624.2	0.69	0.60	0.84
*INHBB*	ILMN_1685714	57	1.1×10^−71^	5.0×10^−71^	136.3	0.64	0.17	0.08
*GLI2*	ILMN_1727577	11	0.00015	0.00031	8.9	NA^8^	NA^8^	NA^8^

1 Number of samples with a GenomeStudio ‘pDetection’ p-value≤0.05.

2 Computed transcript detection p-value.

3 False discovery rate detection p-value.

4 Mean average raw signal.

5 ‘transcript∼preeclampsia’ regression p-value.

6 ‘transcript∼preeclampsia+rs7579169’ regression p-value.

7 ‘transcript∼preeclampsia+rs12711941’ regression p-value.

8 No regression analyses performed.

### Bioinformatic analysis of associated SNPs

Using the UCSC genome browser (Human, Feb. 2009 [GRCh37/hg19]) we conducted bioinformatic analyses on the two significantly associated GWAS SNPs (rs7579169, rs12711941) and the rs7576192 SNP identified by re-sequencing to see if they resided within (1) regulatory elements (histone mark H3K4Me1 or DNase I hypersensitive sites from ENCODE) or (2) transcription factor (TF) binding sites (ChIP-seq data from ENCODE). Additional TF binding site analysis was performed using P-Match [30], [31] and AliBaba 2.1 [30], [32]. Histone marks in the regions of rs7579169 and rs7576192 suggest some promoter/enhancer activity to be present, and highest in human umbilical vein endothelial cell (HUVEC) lines. AliBaba indicated a Sp1 (stimulating protein 1) TF binding site in the presence of the minor ‘T’ allele for rs7579169. This would suggest the minor allele for rs7579169 to be affiliated with higher transcriptional activity/expression. Conversely, AliBaba indicated a Sp1 TF binding site in the presence of the major ‘G’ allele for rs12711941. This would suggest the minor allele for rs12711941 to be affiliated with lower transcriptional activity. No TF binding sites were identified in the presence of the major or minor allele for rs7576192. The rs7579169, rs12711941 and rs7576192 SNPs all reside within regions of low sequence conservation and/or low complexity, suggesting functional importance is low. It is therefore more likely that these SNPs are in LD with an as yet unidentified polymorphism of greater functional significance.

### Norwegian and Finnish replication cohorts

The two GWAS SNPs showing significant association with preeclampsia in the Australian cohort were genotyped in two independent case-control cohorts from Norway (1,134 preeclampsia cases, 2,263 normal pregnancy controls) and Finland (760 preeclampsia cases, 759 normal pregnancy controls). The rs7579169 SNP association for the minor ‘T’ allele was not replicated in the Norwegian (p = 0.29, MAF_(cases)_ = 0.424, MAF_(controls)_ = 0.438) or Finnish (p = 0.60, MAF_(cases)_ = 0.391, MAF_(controls)_ = 0.382) cohorts (Table S4). Similarly, the rs12711941 SNP association for the minor ‘T’ allele was not replicated in the Norwegian (p = 0.35, MAF_(cases)_ = 0.422, MAF_(controls)_ = 0.434) or Finnish (p = 0.50, MAF_(cases)_ = 0.387, MAF_(controls)_ = 0.375) cohorts (Table S4).

## Discussion

The determination of the genetic contributions to risk of preeclampsia has proven difficult. In this first GWAS for preeclampsia we have obtained strong evidence for a risk locus on chromosome 2q14.2 defined by significant genetic association with two intergenic SNPs located within 15 kb of the 3′ terminus of the Inhibin, beta B (*INHBB*) gene. Our subsequent re-sequencing of the *INHBB* locus in a small sample of affected and unaffected individuals from our Australian cohort identified a third intergenic SNP, also residing within 15 kb from the *INHBB* 3′ terminus, to be significantly associated with preeclampsia. While all three intergenic SNPs are in strong LD with each other they are not in LD with any other genotyped SNP within ±250 kb.

Our preliminary bioinformatic and transcriptional profiling analyses have not provided compelling data to implicate these SNP variants and/or genes in preeclampsia etiology, and we did not replicate these significant SNP associations in either Norwegian or Finnish case-control cohorts. While successful replication can provide an important and independent verification of a putative genetic association, which helps to prevent the discovery of spurious associations, failure to replicate in a population different from that used in the initial finding does not necessarily invalidate the original observation. The reasons why true associations may not replicate across independent data sets has received considerable attention over the last five years with genetic heterogeneity, environmental interactions, age-dependent effects, epistasis and inadequate statistical power given as possible reasons [33]–[36]. In this context it is perhaps noteworthy that in our earlier linkage-based positional cloning studies in Australian families where we reported the likely involvement of the activin type 2A receptor (*ACVR2A*) gene [24], [37] and the endoplasmic reticulum aminopeptidase 2 (*ERAP2*) gene [26] in risk of preeclampsia, we were also unable to replicate our gene-specific SNP associations in the same Norwegian case-control cohort as that used in this current study. In the case of *ACVR2A* and *ERAP2* we subsequently were able to demonstrate association with preeclampsia in the Norwegian population using other SNPs in these genes, providing evidence of different allele frequencies and LD patterns at these loci [25], [26]. These data may be consistent with the existence of as yet unidentified/untyped rare risk variants that exhibit different patterns of linkage disequilibrium in our Australian, Norwegian and Finnish population samples.

While we have not presented compelling functional data to implicate any genes at the 2q14.2 locus marked by our SNP associations, we are encouraged by the striking plausibility of the *INHBB* gene as a positional candidate susceptibility gene for preeclampsia. This is supported by a body of substantive biological data that is consistent with the involvement of the activins, inhibins and other members of the TGF-β superfamily in the development of preeclampsia [38]–[45]. It is worth noting that, during pregnancy activins and inhibins are produced in the human endometrium, decidua and placenta and are thought to inactivate matrix metalloproteases in human endometrial stromal cells during decidualization thereby affecting remodeling of the maternal spiral arteries by the invading cytotrophoblasts [46]. Failed remodeling of these vessels is regarded as an early defining event in the pathophysiology of preeclampsia [47]–[49]. The fact that INHBB is biologically connected to ACVR2A leads us to speculate that our positional cloning studies in the Australian Caucasian population, originally using linkage mapping in families and now GWAS in unrelated individuals, have revealed positional candidate genes that define a key pathway involved in susceptibility to preeclampsia. We now propose to focus our efforts on the identification of probable rare and as yet unidentified variants in the inhibins, activins and their receptors as such variation is likely to be critical to the development of preeclampsia in many populations.

## Materials and Methods

### Ethics Statement

#### Australian GWAS cohort

Ethical approval for the recruitment of the Australian women was granted by the RWH Research and Ethics Committees, Melbourne, Australia. Written informed consent was obtained from study participants prior to them being phlebotomized. Permission was also granted from the Australian case-control cohort women to access and examine their medical records in order to confirm/validate Caucasian ancestry and relevant preeclampsia diagnostic criteria. Ethical approval to conduct molecular and statistical analyses of the Australian samples was obtained from the Institutional Review Board (IRB) of the University of Texas Health Science Center at San Antonio (UTHSCSA).

#### Norwegian replication cohort

All HUNT participants provided written informed consent when recruited to the study. Prior approval to use the Norwegian case-control cohort for genetic studies was obtained by the Regional Committee for Medical Research Ethics, Norway and approved by the National Data Inspectorate and The Directorate of Health and Social Welfare. Ethical approval for the molecular and statistical analysis of the Norwegian samples was obtained from the IRB of the UTHSCSA.

#### Finnish replication cohort

All subjects provided a written informed consent. The FINNPEC study protocol was approved by the coordinating Ethics Committee of the Hospital District of Helsinki and Uusimaa. The Southern Finnish participant study was approved by the local ethical review committee at the Helsinki University Hospital. Ethical approval for the molecular and statistical analysis of the Finnish samples was in addition obtained from the IRB of the UTHSCSA.

### GWAS case-control sample population

The Australian case-control cohort of 1,092 unrelated women used in this GWAS included 1,018 women of confirmed Caucasian ancestry (471 preeclampsia cases and 547 normal pregnancy controls) retrospectively ascertained from a larger Australian case-control cohort of 1,774 women that were recruited at the Royal Women's Hospital (RWH), Melbourne, Australia over a five period from 2007 to 2011. The Australian population seen at the RWH in Melbourne is ∼70% Caucasian and for this study the focus was on the recruitment of Caucasian subjects. The additional 74 women were unrelated preeclampsia cases from our Caucasian Australian and New Zealand family cohort that has been described in detail elsewhere [22]–[24], [26].

### Replication case-control sample populations

The most promising SNPs from the Australian GWAS were assessed in two independent case-control cohorts from Norway and Finland.

#### Norway

All Norwegian samples were retrospectively selected from a large multipurpose health survey conducted over a three period from 1995 to 1997 in Nord-Trøndelag County in Norway [50]. More than 65,000 inhabitants participated. The people living in the Nord-Trøndelag County are considered to be representative of the Norwegian population, and are well suited for genetic studies because of their ethnic homogeneity (<3% non-Caucasians) [50], [51]. Information pertaining to all pregnancies and deliveries has been registered in the Medical Birth Registry of Norway (MBRN) since 1968. The MBRN has established formal classifications of different diseases in pregnancy. The unique 11-digit national identification numbers from HUNT2 women participants were cross referenced with the information registries of the MBRN to identify case-control cohorts. The HUNT study population used to study preeclampsia has been described in detail elsewhere [25], [52].

#### Finland

The Finnish patient samples used in this study originate from the Finnish Genetics of Preeclampsia Consortium (FINNPEC) study cohort and the Southern Finland preeclampsia study cohort. FINNPEC is an ongoing multicentre study where DNA samples and data have been collected prospectively at all university hospitals in Finland (i.e. Helsinki, Turku, Tampere, Kuopio and Oulu) from 2008. For each woman with preeclampsia, the next available woman giving birth at the same hospital, with no preeclampsia, is invited as a control. After initial review of hospital records by a research nurse, each diagnosis is confirmed by a study physician based on criteria described below. Information pertaining to the Southern Finnish case-control cohort was obtained from discharge records from the Helsinki University Central Hospital. These records were used to retrospectively identify women with preeclampsia between January 1988 and April 1998 [53]. These women were healthy prior to their first pregnancy with no evidence of renal or autoimmune disease. Blood samples were collected between January 1997 and April 1998 after the index pregnancy [53], [54]. During the same period, blood samples from non-preeclamptic (control) patients who had given birth in the same hospital were also collected.

### Preeclampsia diagnosis

#### Australian GWAS cohort

Preeclampsia diagnosis was determined by qualified clinicians using criteria set by the Australasian Society for the Study of Hypertension in Pregnancy [55], [56], and the Society of Obstetric Medicine of Australia and New Zealand for the management of hypertensive diseases of pregnancy [57]. Women were considered preeclamptic if they were previously normotensive and if they, on at least two occasions six or more hours apart, had after 20 weeks gestation (i) a rise in systolic blood pressure (SBP) of at least 25 mmHg and/or a rise from baseline diastolic blood pressure (DBP) of at least 15 mmHg, or (ii) SBP≥140 mmHg and/or DBP≥90 mmHg. Additionally, significant new onset proteinuric levels were either ≥0.3 g/l in a 24 hour specimen, at least a ‘2+’ proteinuria dipstick reading from a random urine collection or a spot protein∶creatine ratio ≥0.03 g/mmol. Preeclamptic women who also experienced convulsions or unconsciousness in their perinatal period were classified as having eclampsia. Women with pre-existing hypertension or other medical conditions known to predispose for preeclampsia (e.g. renal disease, diabetes, twin pregnancies or fetal chromosomal abnormalities) were excluded. Of the 1,774 unrelated Australian women initially recruited for this study, 1,018 women were of confirmed Caucasian ancestry, meeting our inclusion criteria. Of these, 471 were confirmed, by medical records, as having preeclampsia (cases) and 547 were confirmed as having a normal pregnancy (controls). An additional 74 unrelated preeclamptic (case) women selected for inclusion in our GWAS sample were the probands and/or founders of our previously described 74 preeclampsia families [22]–[24], [26], [27], [37], [58].

#### Norwegian replication cohort

The definition and classification of preeclampsia used for the Norwegian samples was established by the MBRN based on previously reported guidelines [11]. The MBRN definition for preeclampsia was defined as an increase in SBP to at least 140/90 mmHg (or an increase in SBP≥30 mmHg, or in DBP≥15 mmHg from the level measured before the 20^th^ week of gestation), combined with proteinuria (protein excretion of at least 0.3 g per 24 hours or ≥1+ on a dip stick). Based on these diagnostic criteria there were 1,179 women registered with preeclampsia (cases) and 2,358 women with a history of a normal, healthy pregnancy (controls). Of these registered women, blood samples were available for 1,134 cases and 2,263 controls at the HUNT Biobank and included for this study.

#### Finnish replication cohort

Finnish women who suffered a preeclamptic pregnancy and had no medical history of chronic hypertension, type 1 diabetes, or renal disease were eligible for the study as cases. Diagnostic criteria used for the FINNPEC study cohort were SBP≥140 mmHg and/or DBP≥90 mmHg on at least two occasions with new onset proteinuria (≥0.3 g/24 hrs, or ≥0.3 g/L, or in the absence of concurrent quantitative measurement, at least a ‘2+’ or more, or two ‘1+’ proteinuria dipstick readings) after 20 weeks gestation in a previously normotensive woman. Preeclampsia in the Southern Finnish case/control cohort was defined as two SBP/DBP measurements at least 6 hrs apart ≥140/90 mmHg and proteinuria measurement ≥0.3 g in a 24 hour urine collection, or at least a ‘1+’ dipstick reading after 20 weeks gestation [53]. A total of 760 preeclamptic (case) women and 664 control women from the FINNPEC study cohort, and 95 control women from the Southern Finland preeclampsia study cohort were included in this study.

### GWAS genotyping

The isolation of genomic DNA (gDNA) from the Australian case-control blood samples was achieved using Qiagen's Blood & Cell Culture DNA Midi Kit (Qiagen Pty Ltd, Doncaster, VIC, Australia). The individual gDNA samples (n = 1,092) were genotyped using Illumina's Human OmniExpress-12 BeadChip (Illumina Inc., San Diego, CA) containing 731,442 loci derived from phases I, II and III of the International HapMap project [59]–[61]. A total of 200 ng of gDNA (4 µl at 50 ng/µl) for each sample was processed according to Illumina's Infinium HD Assay Ultra protocol. BeadChips were imaged on Illumina's iScan System with iScan Control Software (v3.2.45). Normalization of raw image intensity data, genotype clustering and individual sample genotype calls were performed using Illumina's GenomeStudio software (v2010.2), Genotyping Module (v1.7.4). Illumina's pre-defined genotype cluster boundaries were used to denote SNP genotype cluster positions (HumanOmniExpress-12v1_C.egt). Additionally, genotype clusters for all SNPs of interest were visually inspected (Figures S1 & S2). Genotype assay quality control measures were assessed with Illumina's internal assay performance metrics. Individual SNP loci and individual sample quality control performance measures were assessed using PLINK [29]. Individual SNP loci were excluded, (i) if genotype success rates were <0.95 (n = 4,742); (ii) for deviation from Hardy-Weinberg equilibrium in the control samples with a criterion of p<0.0001 (n = 1,676); (iii) if the observed copies of the minor allele in the population sample (i.e. cases and controls, collectively) was <10 (n = 77,286). This quality control metric equates to a minor allele frequency (MAF) being less than 0.009 (10/1,092); (iv) for any residual non-autosomal or X-linked loci (n = 380 XY-linked loci). Given our female only data set, X-linked loci were retained in our analyses. Individual samples were excluded, (i) if genotype call rates were <0.9 (1 case, 1 control); (ii) if PLINK's sex check to estimate X chromosome inbreeding (homozygosity) rates (F) was ≥0.2 (3 cases, 1 control). For this quality control metric a female call is made if F<0.2 and was conducted to identify probable random genotype error(s); (iii) using PLINK's cryptic relatedness metric to examine the possibility of unknown, distant familial relationships amongst the Australian GWAS sample set by estimating the proportion of alleles shared identical by descent (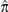). Eight pairs of DNA samples putatively exhibited a distant familial relationship (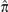
≥0.125), of which 3 cases and 5 controls were excluded from subsequent data analyses. These SNP loci and sample quality control metric thresholds resulted in the passing of 648,175 SNPs to be analyzed in 1,078 unrelated Australian women (538 preeclampsia cases, 540 normal pregnancy controls). The mean (range) genotyping success rate of the quality control filtered data set was 0.9986 (0.9499–1).

### DNA sequencing

Gene-centric and/or conserved intergenic regions flanking prioritized SNPs were sequenced in 96 unrelated Australian samples (48 preeclampsia cases, 48 normal pregnancy controls). These samples were a subset of the final GWAS sample set (n = 1,078) that passed our quality control cleaning. Conserved intergenic regions were identified using the ECR Browser [62]. Genomic DNA sequence reference templates were obtained from the UCSC Genome Bioinformatics database (Human, Feb. 2009 [GRCh37/hg19]). All primers were designed using Primer 3 (v0.4.0) and BLASTed to assess their uniqueness to the human genome. Contiguous primer pairs were designed to overlap by ∼100–150 bp. Standard PCR was performed with 20 ng of gDNA in a 10 µl reaction volume. If standard PCR optimization conditions failed, FailSafe PCR pre-mixes (Epicentre Biotechnologies, Madison, WI) were used in lieu. GeneAmp 9700 thermal cyclers (Life Technologies, Foster City, CA) were used for PCR amplification. PCR amplicons were purified with ExoSAP-IT (USB Corp., Cleveland, OH) according to manufacturers' instructions. Independent sequencing reactions for both the sense and anti-sense strands were performed on the purified PCR amplicons (1 µl) using AB BigDye Terminator v3.1 chemistry (Life Technologies) in a 5 µl reaction volume. Sequence reaction amplification was performed on a GeneAmp 9700 thermal cycler using standard cycling conditions. Amplified sequence products were purified with AB BigDye XTerminator purification kits according to manufacturers' instructions (Life Technologies). Purified sequence reactions were electrophoretically separated on an AB 3730xl DNA Analyzer (Life Technologies). Sequence reaction quality was assessed using Sequencing Analysis software v5.1.1 and sequence variant identification was performed using SeqScape v2.6 (Life Technologies).

### Replication and targeted loci genotyping

Additional genotyping in the Australian cohort and replication genotyping in the Norwegian and Finnish cohorts was performed using Sequenom-based MassArray technology (Sequenom, San Diego, CA). SNP assays were designed using Sequenom's online design tools in conjunction with Assay Designer v4.0. Variant specific PCR and single-base extension primers were supplied by Integrated DNA Technologies (IDT, Coralville, IA). For each sample, 20 ng of gDNA was used and assayed in accordance with the iPLEX Gold Reaction protocol using the MassARRAY Matrix Liquid Handler. Samples were spotted onto a 384-sample SpectroCHIP II using the MassARRAY Nanodispenser RS1000. SpectroCHIPs were loaded into the MassARRAY Analyzer 4 and the nucleotide mass time-of-flight was recorded using SpectroACQUIRE software (v4.0.2.52). Genotype clustering and individual sample genotype calls were generated using Sequenom's TyperAnalyzer (v4.0.5). To assess the accuracy of the GWAS genotypes we re-genotyped our prioritized SNPs back in the Australian GWAS cohort.

### Transcriptional profiling in decidua

Of the 1,078 unrelated Australian women that passed our GWAS quality control decidual basalis tissue was also available from 25 preeclampsia cases and 35 healthy pregnancy controls. These decidual samples were collected at the time of delivery by Caesarean section, from the placental bed by suction curettage, as previously described [63]. Total RNA isolation and quality assessment, and anti-sense RNA (aRNA) synthesis, amplification and purification were performed as previously described [63]. Purified aRNA was hybridized to Illumina's HumanHT-12 v4 Expression BeadChips in accordance with Illumina's Whole-Genome Gene Expression Direct Hybridization assay protocol. All samples were scanned on the Illumina iScan System with iScan Control software (v3.2.45). Illumina's GenomeStudio software (v2010.2), Gene Expression Module (v1.7.0) was used to generate a control summary report to assess assay performance and quality control metrics. One control sample failed the image scan and was subsequently omitted prior to data analysis. The remaining 59 tissue samples yielded high quality expression profile data, without any samples showing a marked reduction in the number of probes detected, in mean average raw signal, or in mean correlation (in raw expression level across probes) with the other samples.

### Data analyses

#### Population structure

To account for potential population structure within the Australian GWAS samples passing quality control (n = 1,078), principal components analysis (PCA) was conducted in R (prcomp) using a subset of quality control filtered SNPs (n = 246,406). The subset of common SNPs (MAF≥0.05) for PCA was generated using PLINK to compute the genotypic correlation (r^2^) between SNP pairs within a 50 SNP window. Each SNP window progressed forward by 5 SNPs prior to re-computing pairwise genotypic correlations. One SNP from a pair of SNPs was excluded if r^2^>0.5. PCA revealed very minimal population structure in the Australian GWAS samples, so principle components correction was not used in the association analysis. The absence of false positive association due to population structure was confirmed by the calculated genomic inflation factor (λ) of 1.002.

#### Genome-wide data analysis

Due to minimal population structure, asymptotic p-values for each of the quality control filtered SNPs (n = 648,175) were computed to assess minor-allele association with the disease trait (i.e. preeclampsia) using PLINK. The Manhattan plot displaying the −log10 transformation of observed p-values was generated using the mhtplot function of the R package ‘gap’. The Q-Q plot depicting −log10 transformations of observed p-values as a function of expected p-values was generated using R base graphics. The asplot function of the R package ‘gap’ was used to generate a regional association plot for loci of interest (±250 kb) based on, recombination rate (HapMap 2006-10_rel21_phaseI+II), PLINK computed pairwise genotypic correlations between all genotyped SNPs in the Australian samples (–ld-window-r2 0) and PLINK generated point-wise, asymptotic association test p-values.

#### Genome-wide multiple testing correction

To attain adjusted genome-wide significant and suggestive thresholds we first imputed sporadic missing genotype data using BEAGLE [64]. An effective number of independent SNP tests across our GWAS data set were approximated using the solid spine of linkage disequilibrium (SSLD) measure implemented in HAPLOVIEW [65], as previously described [66]. An approximated effective number of independent SNP tests were used to calculate modified Bonferroni-adjusted significant and suggestive thresholds. Briefly, juxtaposed chromosome specific SNP windows containing at most 3,000 SNPs were first generated using PLINK. Using HAPLOVIEW, the number of SNP blocks and interblock SNPs were determined with a minimum D′ value of 0.8. Pairwise comparisons of SNPs more than 500 kb apart were ignored. Quality control filtered SNPs that did not satisfy HAPLOVIEW's SSLD default parameters (i.e. MAF<0.01; HWE p<0.001), or were not assigned a chromosomal bp coordinate with the Illumina SNP chip annotation were treated as independent SNPs akin to the interblock SNPs. These additional independent SNPs are herein referred to as ‘residual SNPs’. For each chromosome the sum of SNP blocks, interblock SNPs and residual SNPs approximate the effective number of independent SNP tests. The estimated number of independent SNPs (SNP_INDEP_), specific to the Australian case-control cohort, was used to generate an adjusted target alpha level (0.05/SNP_INDEP_). For this study, the adjusted genome-wide significant and suggestive thresholds were set at 5.11483×10^−7^ (0.05/97,755) and 1.02297×10^−6^ (0.1/97,755), respectively (Table S1).

#### Targeted loci data analysis

Additional association analyses in the Australian cohort and replication association analyses in the Norwegian and Finnish cohorts were performed in PLINK assuming an additive model of gene action. Extremely rare variants (MAF<0.01) were analyzed in PLINK using the conservative Fisher's Exact Test [67].

#### Gene expression data analysis

To further scrutinize sample quality we computed the mean expression signal across all detected probes for each sample independently. We then computed, for each sample, the mean correlation with all other samples across the raw average signals of all detected probes. All 59 samples passing the initial scan were retained. Using the “pDetection” p-values generated by Illumina's GenomeStudio Gene Expression module, and computing the probability that as many or more tissue samples as observed would yield a p-value≤0.05 by chance, expression of 24,647 probes (52.2% of all probes) was significantly detected. The raw expression levels of these probes (after background subtraction using GenomeStudio) were shifted upwards to force positive values (minimum expression level of 1.0 across all samples and detected probes), log2 transformed, and quantile normalized. To investigate whether expressed probes in the identified candidate 2q14.2 region (*INHBB* structural locus ±500 kb) are significantly correlated with preeclampsia, and whether the identified candidate SNPs (rs7579169, rs12711941) are putatively regulatory variants (expression quantitative trait nucleotides), we preformed linear regression analysis, using disease status (preeclampsia or no preeclampsia) and/or SNP genotype (coded additively as the number of copies of the minor allele present in a person) as predictors of fully processed expression level.

## Supporting Information

Figure S1 GenomeStudio genotype cluster plot for rs7579169.
(TIFF)

Figure S2 GenomeStudio genotype cluster plot for rs12711941.
(TIFF)

Table S1 Estimated number of independent GWAS SNPs.
(DOC)

Table S2 Genotypic correlations (r^2^) of HapMap CEU SNPs flanking the strongest associated preeclampsia SNP (rs7579169±200 kb). bp, distance from rs7579169; SNP Chip, SNPs present (yes) or absent (no) on the Human OmniExpress-12 BeadChip used in this study; ^#^ *INHBB* nearGene-5 SNP; *INHBB intronic SNP.
(DOC)

Table S3 Genotypic correlations between rs7576192 and the other 20 re-sequenced *INHBB* locus variants, plus the two GWAS SNPs; rs7579169 and rs12711941.
(DOC)

Table S4 Replication genotyping in Norwegian and Finnish case-control cohorts. Alleles are listed as major/minor. r^2^ denotes the genotypic correlation between rs7579169 and rs12711941.
(DOC)
