# Supplementary figures and images for: Genome-Wide Association Scan Identifies a Risk Locus for Preeclampsia on 2q14, Near the Inhibin, Beta B Gene
Source: PLoS One. 2012 Mar 14;7(3):e33666. doi: 10.1371/journal.pone.0033666 (PMC3303857; doi:10.1371/journal.pone.0033666)

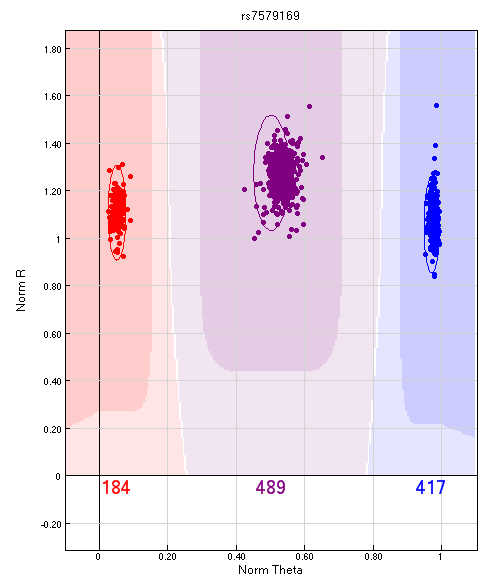

Supplement: Figure S1 — GenomeStudio genotype cluster plot for rs7579169. (TIFF) [file pone.0033666.s001.tif]

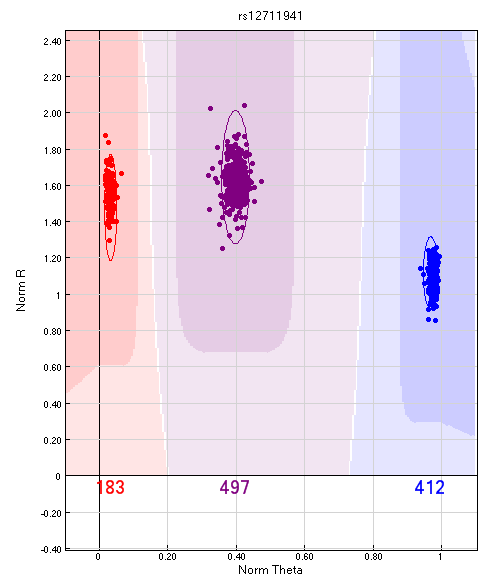

Supplement: Figure S2 — GenomeStudio genotype cluster plot for rs12711941. (TIFF) [file pone.0033666.s002.tif]
