# Supplementary material for: Genome-Wide Association Scan Identifies a Risk Locus for Preeclampsia on 2q14, Near the Inhibin, Beta B Gene
Source: PLoS One. 2012 Mar 14;7(3):e33666. doi: 10.1371/journal.pone.0033666 (PMC3303857; doi:10.1371/journal.pone.0033666)
Supplement: Table S1 — Estimated number of independent GWAS SNPs. (DOC) [file pone.0033666.s003.doc]

**Table S1.** Estimated number of independent GWAS SNPs

| **Chr** | **SNPs** | **SNP blocks** | **Interblock SNPs** | **Residual SNPs** | **SNP_INDEP_** |
| --- | --- | --- | --- | --- | --- |
| 1 | 51,661 | 6,249 | 958 | 124 | 7,331 |
| 2 | 51,299 | 6,456 | 967 | 129 | 7,552 |
| 3 | 42,394 | 5,400 | 806 | 117 | 6,323 |
| 4 | 36,844 | 4,850 | 725 | 87 | 5,662 |
| 5 | 38,360 | 4,898 | 720 | 61 | 5,679 |
| 6 | 43,764 | 4,884 | 680 | 156 | 5,720 |
| 7 | 34,527 | 4,431 | 682 | 112 | 5,225 |
| 8 | 33,881 | 4,286 | 625 | 89 | 5,000 |
| 9 | 29,988 | 3,898 | 626 | 66 | 4,590 |
| 10 | 34,916 | 4,180 | 603 | 109 | 4,892 |
| 11 | 32,766 | 3,971 | 584 | 97 | 4,652 |
| 12 | 31,952 | 4,024 | 618 | 97 | 4,739 |
| 13 | 24,909 | 3,123 | 458 | 55 | 3,636 |
| 14 | 20,916 | 2,709 | 439 | 46 | 3,194 |
| 15 | 19,467 | 2,618 | 486 | 51 | 3,155 |
| 16 | 20,237 | 2,817 | 523 | 52 | 3,392 |
| 17 | 17,745 | 2,502 | 433 | 59 | 2,994 |
| 18 | 19,242 | 2,581 | 474 | 32 | 3,087 |
| 19 | 13,191 | 1,962 | 379 | 46 | 2,387 |
| 20 | 16,464 | 2,259 | 400 | 34 | 2,693 |
| 21 | 9,312 | 1,255 | 240 | 32 | 1,527 |
| 22 | 9,424 | 1,366 | 268 | 106 | 1,740 |
| X | 14,915 | 2,175 | 361 | 49 | 2,585 |
| **Total** | **648,174** | **82,894** | **13,055** | **1,806** | **97,755** |
