# Supplementary material for: Genome-Wide Association Scan Identifies a Risk Locus for Preeclampsia on 2q14, Near the Inhibin, Beta B Gene
Source: PLoS One. 2012 Mar 14;7(3):e33666. doi: 10.1371/journal.pone.0033666 (PMC3303857; doi:10.1371/journal.pone.0033666)
Supplement: Table S2 — Genotypic correlations (r2) of HapMap CEU SNPs flanking the strongest associated preeclampsia SNP (rs7579169±200 kb). bp, distance from rs7579169; SNP Chip, SNPs present (yes) or absent (no) on the Human OmniExpress-12 BeadChip used in this study; # INHBB nearGene-5 SNP; *INHBB intronic SNP. (DOC) [file pone.0033666.s004.doc]

**Table S2.** Genotypic correlations (r^2^) of HapMap CEU SNPs flanking the strongest associated preeclampsia SNP (rs7579169 ± 200 kb). bp, distance from rs7579169; SNP Chip, SNPs present (yes) or absent (no) on the Human OmniExpress-12 BeadChip used in this study; ^#^*INHBB* nearGene-5 SNP; **INHBB* intronic SNP.

| **SNP** | **r^2^** | **bp** | **SNP Chip** |
| --- | --- | --- | --- |
| rs12617570 | 0.021 | -199539 | No |
| rs4848575 | 0.042 | -199015 | No |
| rs4849834 | 0.034 | -198636 | No |
| rs2028148 | 0.04 | -198332 | No |
| rs17050015 | 0.02 | -194138 | No |
| rs17624225 | 0.029 | -193580 | No |
| rs4849835 | 0.021 | -192258 | No |
| rs7602678 | 0.04 | -189069 | No |
| rs2860989 | 0.006 | -188290 | No |
| rs10209713 | 0.004 | -187158 | No |
| rs13382683 | 0.003 | -184826 | Yes |
| rs6706062 | 0.004 | -184026 | No |
| rs17624445 | 0.001 | -181353 | No |
| rs6712456 | 0.004 | -180040 | No |
| rs17624505 | 0.004 | -179103 | No |
| rs11678387 | 0.007 | -178734 | No |
| rs10209939 | 0.029 | -178089 | No |
| rs7340249 | 0.042 | -175775 | Yes |
| rs12618213 | 0.002 | -169551 | Yes |
| rs34520190 | 0.002 | -169015 | Yes |
| rs3961960 | 0.002 | -168998 | Yes |
| rs3891481 | 0.054 | -162372 | No |
| rs12711937 | 0.042 | -159837 | No |
| rs4849836 | 0 | -157837 | No |
| rs6707627 | 0.051 | -151403 | No |
| rs6730199 | 0.038 | -148269 | No |
| rs2018962 | 0.009 | -143522 | No |
| rs1437421 | 0.016 | -142293 | Yes |
| rs3820768 | 0.009 | -139756 | No |
| rs6734438 | 0.029 | -133680 | No |
| rs6542572 | 0.095 | -131416 | Yes |
| rs6542573 | 0.059 | -131252 | Yes |
| rs2278504 | 0.097 | -129747 | No |
| rs934715 | 0.095 | -129447 | No |
| rs3738965 | 0 | -128590 | No |
| rs4849838 | 0.017 | -124420 | No |
| rs6733204 | 0.097 | -124204 | Yes |
| rs12053228 | 0.033 | -123705 | No |
| rs10199109 | 0.097 | -123591 | No |
| rs4848580 | 0.035 | -117007 | Yes |
| rs4077564 | 0.008 | -113883 | No |
| rs6723584 | 0.035 | -106962 | No |
| rs4849842 | 0.018 | -105458 | No |
| rs4849843 | 0.049 | -105288 | No |
| rs6542579 | 0.035 | -103754 | No |
| rs1370379 | 0.051 | -103535 | No |
| rs1370380 | 0.035 | -103229 | No |
| rs11893180 | 0.012 | -99655 | No |
| rs11893187 | 0.035 | -99587 | Yes |
| rs11898472 | 0.03 | -98880 | No |
| rs11690576 | 0.002 | -98324 | Yes |
| rs7588151 | 0.035 | -97483 | No |
| rs7587975 | 0.043 | -95384 | No |
| rs3896632 | 0.01 | -94628 | No |
| rs3889223 | 0.002 | -93692 | No |
| rs11688038 | 0.003 | -91542 | No |
| rs7593660 | 0.035 | -91472 | No |
| rs4100690 | 0.049 | -90763 | No |
| rs13412677 | 0.049 | -88607 | No |
| rs4241156 | 0.036 | -86429 | No |
| rs7557096 | 0.035 | -86081 | No |
| rs7605557 | 0.039 | -85392 | No |
| rs10188430 | 0.035 | -84706 | No |
| rs4849847 | 0.035 | -83594 | No |
| rs3933621 | 0.01 | -83305 | No |
| rs3896630 | 0.004 | -81106 | Yes |
| rs3931841 | 0.051 | -76810 | No |
| rs3931840 | 0.036 | -76777 | Yes |
| rs2289174 | 0.035 | -72240 | No |
| rs2289172 | 0.036 | -72008 | No |
| rs4849849 | 0.036 | -71551 | No |
| rs17050072 | 0.033 | -69412 | No |
| rs17050073 | 0.043 | -69246 | No |
| rs11689112 | 0.036 | -68958 | No |
| rs17050078 | 0.044 | -68511 | No |
| rs17050080 | 0 | -67535 | No |
| rs1065518 | 0.035 | -67135 | No |
| rs10496561 | 0.016 | -65800 | Yes |
| rs6739106 | 0.023 | -64880 | No |
| rs2028147 | 0.002 | -59712 | Yes |
| rs4848586 | 0.003 | -56833 | No |
| rs2043722 | 0.043 | -51813 | Yes |
| rs17050098 | 0.043 | -49368 | No |
| rs7606097 | 0.043 | -48355 | No |
| rs6542585 | 0.044 | -48235 | No |
| rs4849857 | 0.021 | -48202 | No |
| rs11673819 | 0.021 | -47395 | No |
| rs11673820 | 0.009 | -47383 | No |
| rs11673874 | 0.014 | -47313 | Yes |
| rs13018516 | 0.039 | -40940 | Yes |
| rs7563709 | 0.01 | -39888 | No |
| rs934721 | 0.02 | -36655 | Yes |
| rs11892488 | 0.001 | -35885 | No |
| rs934716 | 0.008 | -33417 | No |
| rs7593535 | 0.071 | -31445 | Yes |
| rs11903787 | 0.084 | -29942 | No |
| rs4849864 | 0.015 | -28554 | No |
| rs17625845 | 0.129 | -28393 | Yes |
| rs10207633 | 0 | -28254 | No |
| rs10207828 | 0 | -27837 | No |
| rs6728617 | 0 | -24431 | No |
| rs6728735 | 0 | -24355 | No |
| rs10864990 | 0.024 | -23169 | Yes |
| rs7589138 | 0.017 | -22204 | Yes |
| rs10201826 | 0.023 | -22080 | No |
| rs11890218 | 0.023 | -21873 | No |
| rs11897635 | 0.035 | -17288 | Yes |
| rs7578624^#^ | 0.021 | -15645 | No |
| rs13419301^#^ | 0.036 | -15552 | Yes |
| rs11902591* | 0.03 | -12121 | Yes |
| rs7581178 | 0.036 | -7737 | No |
| rs745723 | 0.067 | -5368 | No |
| rs10175965 | 0.059 | -5243 | Yes |
| rs7576183 | 0.115 | -3861 | Yes |
| rs7576192 | 1 | -93 | No |
| rs1548039 | 0.084 | 797 | Yes |
| rs12617824 | 0.116 | 1239 | No |
| rs7589683 | 0.084 | 2428 | Yes |
| rs11123550 | 0.561 | 4606 | Yes |
| rs12711941 | 0.958 | 5259 | Yes |
| rs6741251 | 0.043 | 5517 | No |
| rs11887035 | 0.004 | 5952 | Yes |
| rs6718024 | 0.627 | 6763 | No |
| rs12471301 | 0.712 | 6907 | No |
| rs13410055 | 0.644 | 6948 | Yes |
| rs2164725 | 0.601 | 7754 | No |
| rs12622286 | 0.627 | 8155 | No |
| rs12616090 | 0.076 | 8369 | Yes |
| rs2861032 | 0.075 | 9171 | No |
| rs2901840 | 0.067 | 9644 | No |
| rs1437422 | 0.001 | 11166 | No |
| rs4076867 | 0.505 | 15121 | Yes |
| rs4366914 | 0.382 | 17531 | Yes |
| rs4128408 | 0.328 | 17893 | No |
| rs10164661 | 0.007 | 18265 | No |
| rs4241157 | 0.339 | 20723 | No |
| rs4511723 | 0.327 | 22885 | No |
| rs12479311 | 0.18 | 22936 | Yes |
| rs6712151 | 0.266 | 25466 | No |
| rs10177102 | 0.33 | 27228 | Yes |
| rs4849871 | 0.021 | 33338 | No |
| rs10168048 | 0.001 | 34168 | No |
| rs10168508 | 0.001 | 34690 | Yes |
| rs13393184 | 0.017 | 36206 | Yes |
| rs13406485 | 0.023 | 36364 | No |
| rs6542592 | 0.157 | 37063 | Yes |
| rs6735848 | 0.017 | 37228 | Yes |
| rs6736460 | 0.05 | 37682 | No |
| rs4076654 | 0.266 | 37700 | No |
| rs6749797 | 0.056 | 37938 | No |
| rs12622427 | 0 | 38829 | No |
| rs10211605 | 0.231 | 40863 | No |
| rs10206874 | 0.126 | 42021 | Yes |
| rs12623934 | 0.02 | 43278 | Yes |
| rs7608411 | 0.007 | 45903 | Yes |
| rs11685966 | 0.114 | 46410 | No |
| rs7562502 | 0 | 47560 | No |
| rs9308780 | 0.093 | 53554 | Yes |
| rs10432608 | 0.114 | 53802 | Yes |
| rs12620947 | 0.003 | 54513 | No |
| rs10170796 | 0.091 | 55660 | No |
| rs4077985 | 0.19 | 56036 | No |
| rs4566372 | 0.057 | 56080 | No |
| rs4456710 | 0.075 | 57764 | Yes |
| rs4849872 | 0.144 | 59442 | No |
| rs7588211 | 0.093 | 60056 | No |
| rs7562511 | 0.063 | 60128 | Yes |
| rs6741645 | 0.099 | 66032 | No |
| rs6731937 | 0.094 | 66096 | Yes |
| rs12477047 | 0 | 68604 | No |
| rs7592778 | 0 | 68939 | Yes |
| rs6729823 | 0 | 69691 | No |
| rs13408457 | 0.07 | 71063 | Yes |
| rs4848595 | 0.107 | 71193 | No |
| rs11890441 | 0.073 | 74528 | Yes |
| rs7563732 | 0.013 | 75632 | No |
| rs12613245 | 0.001 | 75801 | No |
| rs4277528 | 0.067 | 76973 | No |
| rs7581710 | 0.001 | 77057 | No |
| rs10180598 | 0 | 78252 | No |
| rs4449135 | 0.032 | 78959 | Yes |
| rs4300832 | 0.057 | 79217 | No |
| rs7578773 | 0 | 79609 | Yes |
| rs7604844 | 0.003 | 80024 | No |
| rs4583471 | 0.071 | 83906 | No |
| rs4280462 | 0.009 | 84028 | Yes |
| rs10198258 | 0.071 | 84817 | Yes |
| rs10198521 | 0.005 | 85048 | No |
| rs10186456 | 0 | 85387 | Yes |
| rs11903256 | 0.141 | 87839 | No |
| rs6758808 | 0.012 | 88894 | No |
| rs12623001 | 0.021 | 91782 | No |
| rs11123552 | 0.04 | 93697 | No |
| rs4504000 | 0.059 | 96278 | Yes |
| rs11681505 | 0.014 | 96345 | Yes |
| rs11902253 | 0.071 | 97026 | No |
| rs6542598 | 0.023 | 98704 | Yes |
| rs4599120 | 0.003 | 99444 | Yes |
| rs940674 | 0.016 | 100169 | No |
| rs11689518 | 0.035 | 101628 | No |
| rs7572399 | 0.036 | 102749 | No |
| rs7572636 | 0.04 | 102940 | No |
| rs13016912 | 0.014 | 102949 | No |
| rs4849874 | 0.102 | 103741 | No |
| rs7585143 | 0.082 | 104150 | No |
| rs12992152 | 0.094 | 104371 | No |
| rs17050160 | 0.019 | 105150 | Yes |
| rs2140775 | 0.019 | 106073 | No |
| rs2140778 | 0.003 | 107434 | Yes |
| rs6542599 | 0.014 | 110053 | Yes |
| rs11684442 | 0 | 110438 | No |
| rs11684599 | 0.029 | 110763 | No |
| rs17050167 | 0.025 | 110802 | No |
| rs3941990 | 0.005 | 111363 | Yes |
| rs13410508 | 0.021 | 111472 | No |
| rs7558636 | 0.014 | 111489 | No |
| rs4849877 | 0.02 | 111683 | Yes |
| rs6542600 | 0 | 111874 | No |
| rs6711678 | 0.005 | 112947 | No |
| rs4848597 | 0.005 | 113226 | No |
| rs4848598 | 0.012 | 113391 | Yes |
| rs2091255 | 0 | 114637 | No |
| rs11123554 | 0.003 | 115560 | No |
| rs17050177 | 0.021 | 116402 | No |
| rs10204717 | 0.003 | 116736 | Yes |
| rs10204728 | 0.001 | 116758 | No |
| rs940677 | 0.003 | 117275 | Yes |
| rs2310899 | 0.003 | 117427 | Yes |
| rs6757434 | 0 | 117636 | No |
| rs3890780 | 0.003 | 118466 | No |
| rs940678 | 0.021 | 119423 | Yes |
| rs4849879 | 0.026 | 121132 | No |
| rs12466825 | 0.026 | 122504 | No |
| rs12711944 | 0.029 | 122713 | No |
| rs1534100 | 0.026 | 123783 | No |
| rs7575067 | 0.029 | 123834 | No |
| rs7575205 | 0.021 | 123963 | No |
| rs4848601 | 0.02 | 124887 | No |
| rs7571527 | 0.021 | 125566 | No |
| rs12711946 | 0.029 | 126048 | No |
| rs9308781 | 0.001 | 126368 | No |
| rs9789411 | 0.029 | 126385 | No |
| rs9308782 | 0.026 | 126685 | No |
| rs4849884 | 0.026 | 126823 | Yes |
| rs4849887 | 0.026 | 126998 | No |
| rs12616822 | 0.029 | 127416 | No |
| rs10864991 | 0.026 | 127611 | No |
| rs11123556 | 0.026 | 127872 | No |
| rs10179592 | 0.029 | 128444 | No |
| rs7574050 | 0.004 | 128969 | Yes |
| rs7557650 | 0.004 | 129008 | No |
| rs7574164 | 0.004 | 129047 | No |
| rs17050219 | 0.004 | 139004 | Yes |
| rs874816 | 0.003 | 146347 | Yes |
| rs4848605 | 0.026 | 149436 | Yes |
| rs11123558 | 0.004 | 151004 | No |
| rs4849900 | 0.001 | 153525 | Yes |
| rs10188887 | 0.013 | 153553 | Yes |
| rs6707882 | 0.025 | 155807 | No |
| rs6711242 | 0.013 | 155844 | No |
| rs9808249 | 0.007 | 158172 | No |
| rs11883586 | 0.002 | 158516 | No |
| rs11890696 | 0.016 | 159406 | No |
| rs4848606 | 0.013 | 160333 | No |
| rs7585627 | 0.013 | 162479 | Yes |
| rs7572032 | 0.013 | 162643 | No |
| rs11884709 | 0 | 164466 | No |
| rs17050244 | 0.002 | 164506 | No |
| rs4848607 | 0.001 | 165600 | Yes |
| rs940681 | 0.001 | 166009 | No |
| rs940683 | 0.002 | 166832 | No |
| rs17050251 | 0.001 | 168753 | No |
| rs13413159 | 0.002 | 168981 | No |
| rs17050252 | 0.002 | 169288 | No |
| rs13406402 | 0 | 170238 | No |
| rs13422372 | 0 | 170404 | No |
| rs2311300 | 0.002 | 170695 | No |
| rs2311301 | 0.002 | 170708 | No |
| rs2311302 | 0 | 170983 | Yes |
| rs17050253 | 0.002 | 171268 | Yes |
| rs11903416 | 0.014 | 172542 | Yes |
| rs13427257 | 0.004 | 172801 | Yes |
| rs17050256 | 0.029 | 173364 | No |
| rs17050257 | 0.068 | 174044 | Yes |
| rs12473939 | 0.014 | 174730 | No |
| rs7591931 | 0.008 | 175027 | No |
| rs7594811 | 0.008 | 175451 | No |
| rs7340400 | 0.008 | 175723 | No |
| rs7340408 | 0.008 | 175999 | No |
| rs13397485 | 0.008 | 176925 | No |
| rs10205124 | 0.003 | 177325 | No |
| rs6541721 | 0.008 | 177985 | No |
| rs6541722 | 0.01 | 178049 | Yes |
| rs6758414 | 0.043 | 178361 | Yes |
| rs10164570 | 0.008 | 179184 | No |
| rs6541723 | 0.002 | 183113 | No |
| rs6541724 | 0.001 | 183417 | No |
| rs6721532 | 0.026 | 183636 | Yes |
| rs6721654 | 0.001 | 183787 | No |
| rs13432455 | 0.002 | 184570 | No |
| rs2140779 | 0.067 | 184595 | Yes |
| rs17389324 | 0.002 | 185116 | Yes |
| rs17050267 | 0.002 | 185659 | Yes |
| rs6726987 | 0.001 | 186243 | No |
| rs4849902 | 0.001 | 186424 | No |
| rs11122800 | 0.03 | 187480 | No |
| rs2311597 | 0.019 | 187647 | No |
| rs17050272 | 0.004 | 188316 | Yes |
| rs11679833 | 0.036 | 188558 | No |
| rs954244 | 0.004 | 191107 | No |
| rs2030745 | 0.006 | 191158 | No |
| rs2030746 | 0.016 | 191364 | Yes |
| rs12617659 | 0.019 | 191635 | Yes |
| rs12617662 | 0.024 | 191654 | No |
| rs12617782 | 0.019 | 192015 | No |
| rs6706968 | 0.014 | 192145 | No |
| rs940688 | 0.006 | 192876 | No |
| rs11677557 | 0.019 | 199623 | No |
