# Supplementary material for: Genome-Wide Association Scan Identifies a Risk Locus for Preeclampsia on 2q14, Near the Inhibin, Beta B Gene
Source: PLoS One. 2012 Mar 14;7(3):e33666. doi: 10.1371/journal.pone.0033666 (PMC3303857; doi:10.1371/journal.pone.0033666)
Supplement: Table S3 — Genotypic correlations between rs7576192 and the other 20 re-sequenced INHBB locus variants, plus the two GWAS SNPs; rs7579169 and rs12711941. (DOC) [file pone.0033666.s005.doc]

**Table S3.** Genotypic correlations between rs7576192 and the other 20 re-sequenced *INHBB* locus variants, plus the two GWAS SNPs; rs7579169 and rs12711941.

| **Variant** | **bp** | **r^2^** |
| --- | --- | --- |
| ss469271203 | 121101331 | 0.004808 |
| ss469271213 | 121101394 | 0.008249 |
| rs7578624 | 121102479 | 0.015614 |
| rs13419301 | 121102572 | 0.024444 |
| ss469271204 | 121105292 | 0.006936 |
| rs11902591 | 121106003 | 0.015472 |
| rs4328642 | 121106850 | 0.017738 |
| ss469271205 | 121106946 | 0.002744 |
| ss469271217 | 121107784 | 0.00279 |
| ss469271206 | 121107831 | 0.002744 |
| rs45624437 | 121108182 | 0.008063 |
| ss469271207 | 121108506 | 0.002793 |
| ss469271208 | 121108585 | 0.003249 |
| rs57802235 | 121109444 | 0.025234 |
| ss469271209 | 121109737 | 0.003235 |
| rs10183524 | 121109878 | 0.016582 |
| ss469271210 | 121110151 | 0.003874 |
| ss469271218 | 121116483 | 0.001605 |
| ss469271211 | 121116625 | 0.002721 |
| ss469271212 | 121116672 | 0.001615 |
| rs7576192 | 121118031 | 1.0 |
| rs7579169 | 121118124 | 0.978015 |
| rs12711941 | 121123383 | 0.922089 |
