# Supplementary material for: Genome-Wide Association Scan Identifies a Risk Locus for Preeclampsia on 2q14, Near the Inhibin, Beta B Gene
Source: PLoS One. 2012 Mar 14;7(3):e33666. doi: 10.1371/journal.pone.0033666 (PMC3303857; doi:10.1371/journal.pone.0033666)
Supplement: Table S4 — Replication genotyping in Norwegian and Finnish case-control cohorts. Alleles are listed as major/minor. r2 denotes the genotypic correlation between rs7579169 and rs12711941. (DOC) [file pone.0033666.s006.doc]

**Table S4.** Replication genotyping in Norwegian and Finnish case-control cohorts. Alleles are listed as major/minor. r^2^ denotes the genotypic correlation between rs7579169 and rs12711941.

| **SNP** | **Alleles** | **Norway** | | | | | **Finland** | | | | |
| --- | --- | --- | --- | --- | --- | --- | --- | --- | --- | --- | --- |
|  |  | **MAF_(cases)_** | **MAF_(controls)_** | **HWEp** | **P-value** | **r^2^** | **MAF_(cases)_** | **MAF_(controls)_** | **HWEp** | **P-value** | **r^2^** |
| rs7579169 | C/T | 0.4239 | 0.4375 | 0.8615 | 0.2908 | 0.945 | 0.3912 | 0.3819 | 0.9136 | 0.6016 | 0.853 |
| rs12711941 | G/T | 0.4215 | 0.4336 | 0.7796 | 0.3458 |  | 0.3869 | 0.3750 | 0.9563 | 0.5036 |  |
